# Supplementary figures and images for: Does the introduction of a cobot change the productivity and posture of the operators in a collaborative task?
Source: PLoS One. 2023 Aug 9;18(8):e0289787. doi: 10.1371/journal.pone.0289787 (PMC10411803; doi:10.1371/journal.pone.0289787)

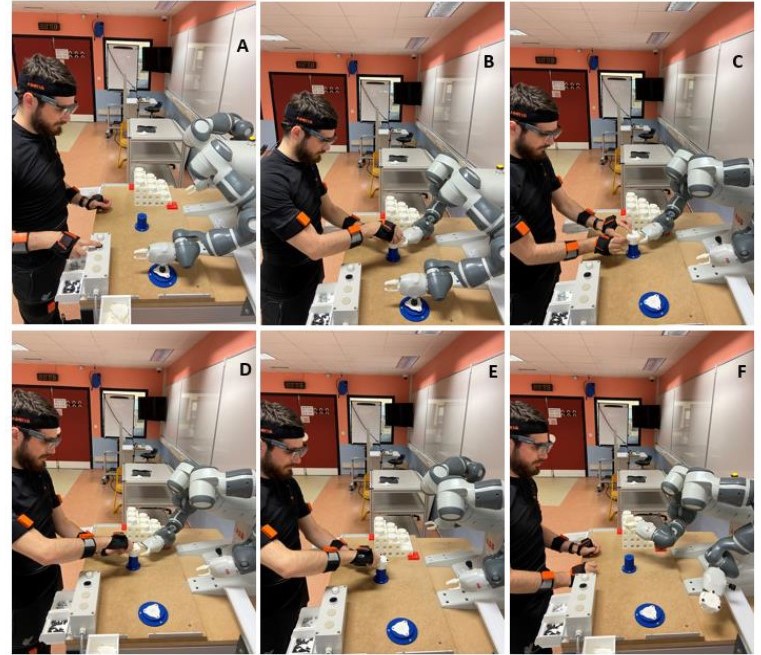

Supplement: S1 Appendix — Here a participant is working with the cobot co-worker. A- Participant press the button on the transmitter; B- Participant inserts an SFP product into the fairing; C- He inserts the first nut; D- Participant screws for the second time; E- He screws for the third time; F- Cobot co-worker evacuates the product and brings the next fairing to the central base. The individual in this manuscript has given written informed consent (as outlined in PLOS consent form) to publish these case details. (TIF) [file pone.0289787.s001.tif]
