# Supplementary material for: Does the introduction of a cobot change the productivity and posture of the operators in a collaborative task?
Source: PLoS One. 2023 Aug 9;18(8):e0289787. doi: 10.1371/journal.pone.0289787 (PMC10411803; doi:10.1371/journal.pone.0289787)
Supplement: Attachment — Submitted filename: Response to Reviewers.docx [file pone.0289787.s003.docx]

Editor-in-chief, *Plos One*

Object: Revision of the paper PONE-D-22-15982

Villers-lès-Nancy, May 30 2023

Dear Editor-in-chief,

Many thanks to give us the possibility to submit a new revised version of our manuscript entitled *“Does the introduction of a cobot change the productivity and posture of the operators in a collaborative task?”.*

We have carefully read the reviewers’ comments and modified the manuscript accordingly. Attached is a detailed, point-by-point response to comments.

We hope this new version will be considered satisfactory and are looking forward to hearing from the Editorial Board.

Yours sincerely.

**Journal requirements:**

- Comment 1:

Please review your reference list to ensure that it is complete and correct. If you have cited papers that have been retracted, please include the rationale for doing so in the manuscript text, or remove these references and replace them with relevant current references. If you need to cite a retracted article, indicate the article’s retracted status in the References list and also include a citation and full reference for the retraction notice.

*Author’s comment:*

We checked the reference list in the manuscript. We removed reference 32 (Richer N, Lajoie Y. Cognitive task modality influences postural control during quiet standing in healthy older adults. Aging Clin Exp Res. 2019;31: 1265–1270. doi:10.1007/s40520-018-1068-9), which was a duplicate of reference 19.

After checking the reference list, we did not find any retracted papers in this list. In your comment, you mentioned retracted papers. If you can provide us with the titles or authors of these papers, we will be able to modify or remove the corresponding references accordingly. Thank you in advance.

- Comment 2:

Any changes to the reference list should be mentioned in the rebuttal letter that accompanies your revised manuscript.

*Author’s comment:*

We have not removed any papers from the initial manuscript. However, we added nine articles based on the comments in the previous review. Here are the nine new articles and references that have been cited:

- 3. Jain R, Meena ML, Sain MK, Dangayach GS. Impact of posture and upper-limb muscle activity on grip strength. International Journal of Occupational Safety and Ergonomics. 2019;25: 614–620. doi:10.1080/10803548.2018.1501972
- 4. Roman-Liu D, Tokarski T. Upper limb strength in relation to upper limb posture. International Journal of Industrial Ergonomics. 2005;35: 19–31. doi:10.1016/j.ergon.2004.07.002
- 11. Roveda L, Maggioni B, Marescotti E, Shahid AA, Maria Zanchettin A, Bemporad A, et al. Pairwise Preferences-Based Optimization of a Path-Based Velocity Planner in Robotic Sealing Tasks. IEEE Robot Autom Lett. 2021;6: 6632–6639. doi:10.1109/LRA.2021.3094479
- 17. Caroly S, Major M-E, Probst I, Molinié A-F. Le genre des troubles musculo-squelettiques: Interventions ergonomiques en France et au Canada. Travail Genre Sociétés. 2013;29: 49–67. doi:10.3917/tgs.029.0049
- 27. Baguley T. Understanding statistical power in the context of applied research. Applied Ergonomics. 2004;35: 73–80. doi:10.1016/j.apergo.2004.01.002
- 36. Rustum R, Adeloye AJ. Replacing Outliers and Missing Values from Activated Sludge Data Using Kohonen Self-Organizing Map. J Environ Eng. 2007;133: 909–916. doi:10.1061/(ASCE)0733-9372(2007)133:9(909)
- 54. Lamon E, De Franco A, Peternel L, Ajoudani A. A Capability-Aware Role Allocation Approach to Industrial Assembly Tasks. IEEE Robot Autom Lett. 2019;4: 3378–3385. doi:10.1109/LRA.2019.2926963
- 55. Bernar A, Prevot C, Legardeur J, Chanal H. Comment favoriser la transition vers l’industrie 4.0 dans le secteur textile-habillement : étude de cas du déploiement d’un cobot au sein de l’entreprise Petit Bateau. 18ème Colloque national S.mart. Carry le Rouet; 2023.
- 56. Maurice P, Allienne L, Malaise A, Ivaldi S. Ethical and Social Considerations for the Introduction of Human-Centered Technologies at Work. 2018 IEEE Workshop on Advanced Robotics and its Social Impacts (ARSO). Genova, Italy: IEEE; 2018. pp. 131–138. doi:10.1109/ARSO.2018.8625830
- 57. Bao SS, Kapellusch JM, Merryweather AS, Thiese MS, Garg A, Hegmann KT, et al. Relationships between job organisational factors, biomechanical and psychosocial exposures. Ergonomics. 2016;59: 179–194. doi:10.1080/00140139.2015.1065347

**Response to Reviewer #2**

- Comment 1:

*Reviewer’s comment:*

The paper can be now accepted. All the concerns have been properly addressed in the revised paper and in the reply to review file.

*Author’s comment:*

Thank you for your comment.

**Response to Reviewer #3**

- Comment 1:

*Reviewer’s comment:*

Thanks for making changes. All the comments raised by reviewer are addressed by the authors in this version.

*Author’s comment:*

Thank you for your comment.

**Response to Reviewer #4**

- Comment 1:

*Reviewer’s comment:*

The authors should send the paper to a native editor to edit the English.

*Author’s comment:*

The paper has been submitted and was reviewed by a native American speaker to check and modify the English of the paper.

- Comment 2:

*Reviewer’s comment:*

In Table 1, it’s not clear what the authors mean by “a the condition with the highest value.”, “b the condition with lower value than a.”… This is not clear at all. The same is in table 2, 3 and 4.

*Author’s comment:*

To clarify the interpretation of the Tables, we have modified the coding to indicate the differences between the conditions.

We used the following three symbols (‡, § and #) with the companying legend: “‡, § and #: For a line, identical symbols indicate no difference between conditions, while different symbols indicate significant difference between conditions.” (example for Table 1 (p.15)).

- Comment 3:

*Reviewer’s comment:*

The added chapter “A theorical method to apply to a more ecological context” does add nothing significant to the paper and only points out one limitation. Instead, the authors should add a part in which comment on the limitations of the study they performed and point out further research directions.

*Author’s comment:*

We replaced this section by highlighting the main limitation of the study, which is that it was conducted in a laboratory setting. We conclude this section by discussing future research that would be valuable to extend the findings of this study in a more ecological situation (p.31-32).

- Comment 4:

*Reviewer’s comment:*

There are only minor aspects:

1.- Line 67: There are no examples of users’ preferences, only three dots.

2.- Line 92: Correct the English in the phrase: “To our knowledge, no study interested to compare a human-human collaboration and a human-robot collaboration for the same task about the production performance or the operators’ health or quality of the interactions”.

3.- Line 95. Correct the English of the last paragraph.

4.- Line 104: Correct the English: “Thirty-four volunteers participated to (in) the study,” I’m going to stop pointing out problems in the English.

*Author’s comment:*

We completed the space of the three dots by adding examples (p.4 l.68). All the English modifications were made with a proofreading by a native English speaker.
